# Synthesis and applications of nanoporous perovskite metal oxides

**DOI:** 10.1039/c7sc03920d

**Published:** 2018-04-02

**Authors:** Xiubing Huang, Guixia Zhao, Ge Wang, John T. S. Irvine

**Affiliations:** a Beijing Key Laboratory of Function Materials for Molecule & Structure Construction , School of Materials Science and Engineering , University of Science and Technology Beijing , Beijing , 100083 , China . Email: gewang@mater.ustb.edu.cn; b Laboratory of Industrical Chemistry , Ruhr-University Bochum , 44780 , Bochum , Germany; c School of Chemistry , University of St Andrews , St Andrews , KY16 9ST , UK . Email: jtsi@st-andrews.ac.uk

## Abstract

Perovskite-type metal oxides have been widely investigated and applied in various fields in the past several decades due to their extraordinary variability of compositions and structures with targeted physical and chemical properties (*e.g.*, redox behaviour, oxygen mobility, electronic and ionic conductivity). Recently, nanoporous perovskite metal oxides have attracted extensive attention because of their special morphology and properties, as well as superior performance. This minireview aims at summarizing and reviewing the different synthesis methods of nanoporous perovskite metal oxides and their various applications comprehensively. The correlations between the nanoporous structures and the specific performance of perovskite oxides are summarized and highlighted. The future research directions of nanoporous perovskite metal oxides are also prospected.

## Abstract 2

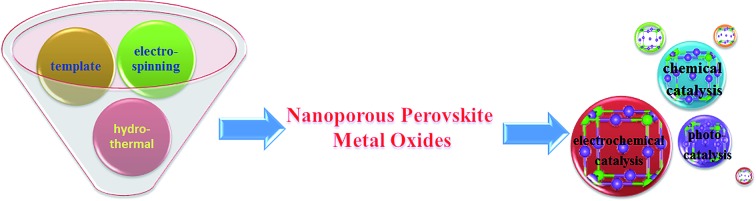
Perovskite-type metal oxides have been widely investigated and applied in various fields in the past several decades due to their extraordinary variability of compositions and structures with targeted physical and chemical properties (*e.g.*, redox behaviour, oxygen mobility, electronic and ionic conductivity).

## 1. Introduction

The perovskite oxides are a well-known family with the general formula of ABO_3_, where A usually stands for an alkaline-earth or rare-earth metal cation, occupying the 12-fold coordinated cub-octahedral cages of the oxygen sub-lattice, and B designates a transition-metal cation (*e.g.*, Mn, Co, Fe, Ni, Cu, Ti) surrounded by six oxygen atoms in an octahedral coordination.^1^ Actually, distortions widely exist in the perovskite structure as a result of subtleties of size ratios on different sites, as well as multiple A or B cations with different sizes and valences, which can be displayed as oxygen nonstoichiometry including both oxygen deficiency and oxygen excess. In addition, perovskite oxides offer great flexibility in redox active sites, oxygen vacancies and physic-chemical properties with tuneable compositions due to the numerous possible substitutions at both A and B sites. Perovskite oxides may also be arranged as double perovskite^2^,^3^ or layered perovskite^4^ based on the composition or crystal structure. Perovskite oxides have great importance and wide applications in many fields as a result of their various compositions and structure, excellent thermal stability, redox behaviour, oxygen mobility, and electronic & ionic conductivity.^1^,^5^ Besides their composition and structure, the morphology of perovskite oxides also has a great influence on their physicochemical properties.

Since the advent of mesoporous silica (*e.g.*, MCM-41 in 1992 (ref. 6) and SBA-15 in 1998 (ref. 7)), nanoporous materials have attracted remarkable interest because of their excellent physicochemical properties, such as possessing a high surface area and large numbers of surface active sites.^8^,^9^ Although numerous methods have been developed to prepare nanoporous materials *via* the design of particle size, morphology, composition, surface and porosity of the nanostructured materials, the preparation of nanoporous materials with multi-metallic oxides is still challenging.^10^,^11^ Compared with silicon precursors which have a slow reaction rate and usually require an acid or basic catalyst for hydrolysis, most metal oxide precursors are usually more reactive, and as a result, the phase separation between organic and inorganic components in metal precursor systems is limited. Besides, the need of calcination at high temperatures to obtain pure phase usually conflicts with the poor thermal stability of nanoporous structures of metal oxides. Applying nanoporous design strategies to perovskite oxides may bring in novel properties and excellent performance in various applications. To the best of our knowledge, there is no comprehensive review about the synthesis and applications of nanoporous perovskite oxides. The current minireview provides a comprehensive summary on the synthesis and applications of nanoporous (*e.g.*, mesoporous (2–50 nm), macroporous (>50 nm) or mixed mesoporous/macroporous) perovskite oxides. The first part presents the synthesis of nanoporous perovskite oxides by various methods, such as soft-template, hard-template, colloid-crystal-template, electrospinning and hydrothermal methods. The second part summarizes the applications of nanoporous perovskite oxides in chemical catalysis, electrochemical catalysis, photocatalysis, and some other fields. At the end of the review, we provide a summary and outlook on this field of research on nanoporous perovskite oxides.

## 2. Synthesis of nanoporous perovskite oxides

In this part, several major synthesis methods (*e.g.*, soft-template, hard-template, colloidal-crystal-template, electrospinning, and hydrothermal methods) for the preparation of nanoporous perovskite oxides are surveyed.

### 2.1 Soft-template method

The use of soft templates (cationic surfactants such as alkyltrimethylammonium surfactants;^12^ anionic surfactants such as C_16_H_33_SO_3_H;^12^ non-ionic surfactant such as block copolymers Pluronic P123 and F127 (ref. 7)) has been widely reported in the synthesis of nanoporous materials, such as mesoporous silica (*e.g.*, MCM-41,^6^ SBA-15 (ref. 7)), mesoporous single metal oxide (*e.g.*, TiO_2_, Al_2_O_3_, SnO_2_, ZrO_2_, Ta_2_O_5_)^13^ with the assumption that the organic templates can be self-assembled and removed after calcination. Direct co-condensation strategies with soft templates are usually adopted, in which the evaporation-induced self-assembly (EISA) approach is one of the most efficient techniques for synthesizing mesoporous oxides. The mechanism of the EISA approach is divided into four steps:^14^ (a) preparation of initial sol homogeneously containing the soft templates and the inorganic precursors with appropriate stoichiometry; (b) evaporation of solvents associated with dip-coating process induced the progressive concentration above the CMC (critical micelle concentration) and self-assembly of inorganic precursors into micelles with poorly condensed network; (c) equilibration of the film with its environment and the final mesostructured adjustment with further inorganic condensation; (d) a thermal treatment induced pre-consolidation, template removal, and network crystallization.

Even though the EISA approach has been widely used for synthesizing mesoporous single-metal oxides, it has been rarely reported as being used for the synthesis of mesoporous perovskite oxides. In order to obtain mesoporous perovskite oxides with pure phases, the cations of the starting gel should be homogeneously mixed in molecular scale during the whole process. However, the heterogeneity in the solubility of the non-volatile components during the solvent evaporation process usually leads to phase separation or secondary phases in the as-prepared samples. In addition, due to the lower temperature for the decomposition of the organic surfactants used in the EISA method than that for the crystallization of perovskite oxides, the mesostructures will collapse as a result of the lack of support at high calcination temperatures, while low calcination temperatures would result in the formation of amorphous phases or impurities. Even though these problems exist, there are still a few reports on the preparation of mesoporous perovskite oxides using the EISA method.^15^–^17^ In 2004, Grosso *et al.* used a semi-commercial organic template (KLE3739, PBH_79_-*b*-PEO_89_) to synthesize mesoporous perovskite oxide films (*e.g.*, SrTiO_3_).^15^ Later, Brezesinski *et al.* adopted the EISA method of associated dip-coating onto a polar substrate using several amphiphilic block copolymers with high thermal stability, such as polyisobutylene-*block*-poly(ethylene oxide), poly(ethylene-*co*-butylene)-*block*-poly(ethylene oxide), to successfully prepare several nanoporous perovskite oxide films (*e.g.*, NaTaO_3_,^18^ BiFeO_3_ (ref. 19)) with 3D honeycomb structures.

Even though the EISA method associated with dip-coating is able to prepare nanoporous perovskite oxides, this method is usually complex and limited to small scale synthesis. Recently, some researchers applied a modified EISA method using precipitant or chelating agents (*e.g.*, citric acid,^16^ urea,^20^,^21^ acetic acid^22^,^23^) to prepare nanoporous perovskite oxides without associating the dip-coating technique. The addition of these chelating agents would lead to a more homogeneous solution and better cation dispersion during the evaporation process but also affected the self-assembly of surfactants and their interaction with metal ions. As a result, using these modified EISA methods, hierarchically nanoporous perovskite oxides can be obtained, including La_1.7_Ca_0.3_Ni_1–*x*_Cu_*x*_O_4_,^16^ La_0.5_Sr_0.5_Co_0.5_Fe_0.5_O_3_,^20^ Sm_0.5_Sr_0.5_CoO_3_,^21^ La_0.68_Ca_0.30_Mn_1.02_O_3–*δ*_,^22^ BaTiO_3_ (ref. 23 and 24) and SrTiO_3_.^25^,^26^

There are still a few papers using the soft-template self-assembly method to prepare highly ordered mesoporous perovskite oxides, majorly based on titanates due to the strong hydrolysis ability of titanium precursors. For example, BaTiO_3_ perovskite oxide with mesostructure inside the crystallites was directly synthesized from the solution *via* a simple sol-precipitation process with cationic surfactant cetyltrimethylammonium chloride (C_16_TMAC).^27^ Yan *et al.* reported a highly ordered mesoporous ZnTiO_3_ with large surface area, large pore volume, and narrow pore size distribution by a sol–gel process combined with EISA in ethanol using Pluronic F127 as structure directing agents.^28^

### 2.2 Hard-template method

The nanoporous hard-template method, also called nanocasting or repeated templating method, is used for the preparation of novel nanostructured materials, wherein porous templates such as mesoporous silica or mesoporous carbon are used to load the metal precursors, followed then by calcination and the removal of the template using acid or alkaline etching. Various kinds of nanoporous materials (*e.g.*, single metal oxides, metal sulfide) have been prepared *via* the hard-template method and detailed summaries can be found in reviews by Zhao and Schüth.^29^–^31^

**Fig. 1:**
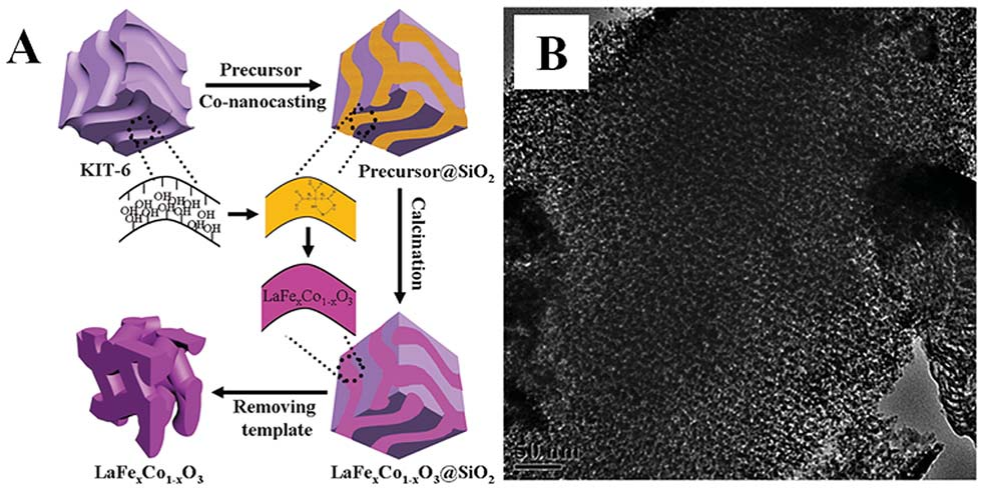
(A) Synthesis process of the mesoporous LaFe_*x*_Co_1–*x*_O_3_ perovskite oxides by co-nanocasting process using mesoporous KIT-6 as a hard template, (B) the as-prepared mesoporous LaFeO_3_. Reproduced with permission from ref. 34. Copyright© 2013, Royal Society of Chemistry.

Over the past decade, the hard-template method has also been used to prepare nanoporous perovskite oxides, including mesoporous LaFeO_3_ or LaNiO_3_ using SBA-15 as a template,^32^,^33^ and LaFe_*x*_Co_1–*x*_O_3_ using KIT-6 as a template.^34^ Due to the use of multi-metal precursors for perovskite oxides, there are some differences between the hard-template methods for perovskite (*e.g.*, addition of chelating agents in the precursor mixture) and single metal oxides. As shown in Fig. 1A, mesoporous silicas (*e.g.*, SBA-15, KIT-6 and MCM-48) or mesoporous carbons (*e.g.*, CMK-1, CMK-3) are usually used as the “hard templates”,^31^,^35^ in which a homogeneous metal salt precursor solution with desired stoichiometry mixed with chelating agents (*e.g.*, citric acid), was prepared to infiltrate the mesoporous support, followed by calcination. Finally, mesoporous perovskite oxides with ordered mesoporous structure and high specific surface area are obtained after the removal of silica by NaOH or HF aqueous solution. The TEM image of mesoporous perovskite oxide is shown in Fig. 1B.

Due to the existence of complex interactions (*e.g.*, hydrogen bonding, coordination, and van der Waals) between the silica and filtrated metal ion precursors, it is usually difficult to fill the mesoporous silica completely at one time, requiring longer periods of time to see a complete impregnation of metal precursors. In addition, there are usually some big perovskite oxide particles outside the pores of mesoporous silica. Therefore, several modified methods have been developed to improve the impregnation of metal precursors and minimize the loading of outside pores, such as functionalization of the mesoporous silica templates with organic groups (*e.g.*, –CH=CH_2_).^36^ However, these reported mesoporous perovskite oxides using mesoporous silicas as a hard template are limited to a few types, mainly with the composition of LaB_1–*x*_B′_*x*_O_3_ (B, B′ = Mn, Co, Fe, Ni) due to the low temperature required for the formation of pure-phased perovskite structure. In particular, the addition of chelating agents such as citric acid into the metal nitrate precursor solutions is of great importance to obtain pure-phased perovskite oxides at relatively low calcination temperatures. Another disadvantage of the hard-template method is that it is difficult to completely drain the silica using NaOH or HF solution, and the silica residue may affect the properties and performance,^32^,^37^ while using mesoporous carbon as a hard template could avoid such problems since the carbon template can be completely removed by calcination at high temperature. For example, high-specific-surface-area LaFe_1–*x*_Co_*x*_O_3_ perovskite oxides were synthesized by nanocasting in a micro-mesoporous carbon, which was replicated from a silica Aerosil 200.^38^ During the calcination process at 800 °C under air, the inorganic precursors are transformed to perovskite oxide nanoparticles simultaneously with the decomposition of the carbon by oxidation. However, there are also some problems using mesoporous carbon as a template, such as poor wetting of the pore walls by the aqueous precursor solution and a low decomposition temperature.

The main problem with the hard-template method is the incomplete filling by conventional impregnation method and the possible formation of perovskite particles outside the pores. Therefore, more facile methods need to be further developed. The double-solvent method using large amount of hydrophobic solvent and pore volume equal aqueous metal precursor solution may provide an effective strategy for better infiltration of metal precursors into the mesoporous structures owing to the high interfacial tension.^39^ Surface modification of silica with various functional groups on the internal or external surfaces would further enhance the impregnation of metal precursors due to the interactions between metal precursors and functional groups.^40^

### 2.3 Colloidal-crystal-template method

**Fig. 2:**
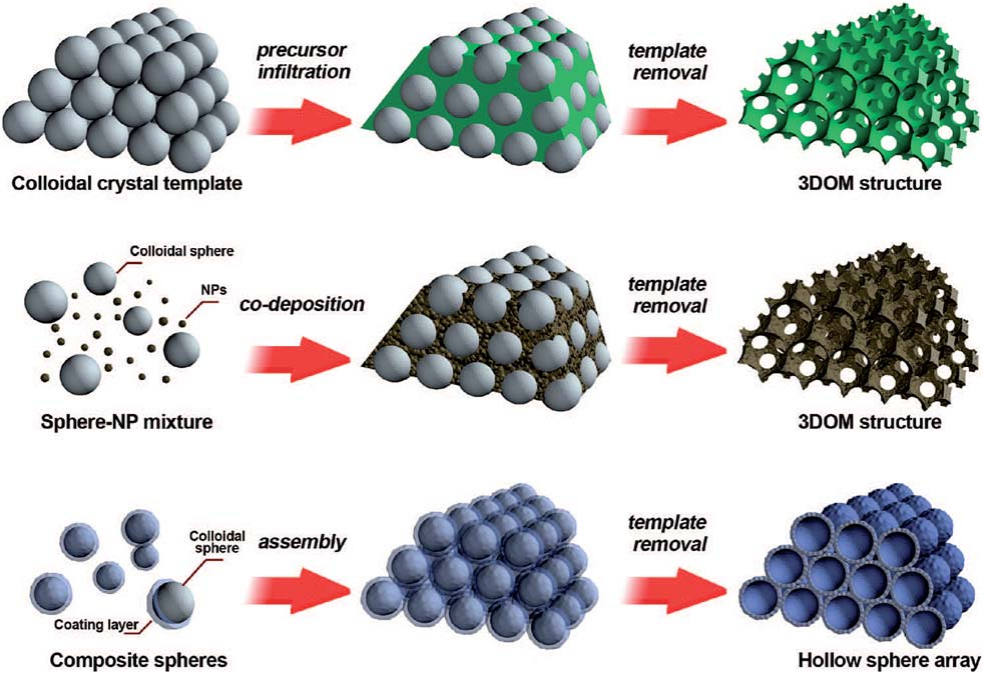
Three methods of preparing periodic macroporous structures by colloidal-crystal-template method. (Top) A preformed colloidal crystal is infiltrated with precursor material which is processed to form the 3DOM structure after removal of the template. (Middle) Uniform templating spheres and nanoparticles (NPs) are co-deposited to form a 3DOM structure after template removal. (Bottom) Core–shell structures are assembled into periodic arrays, forming close-packed hollow shells. Reprinted with permission from ref. 44. Copyright© 2008, American Chemical Society.

The colloidal-crystal-template method is another common synthesis route which is widely used for the preparation of nanoporous perovskite materials with three-dimensionally ordered macropores. The usage of organic polymer spheres (*e.g.*, polystyrene (abbr. PS),^41^,^42^ poly(methyl methacrylate) (abbr. PMMA)^43^) templates to prepare porous inorganic materials with pore sizes ranging from nano- to micrometers has proven very successful. Three methods of preparing periodic macroporous structures by colloidal-crystal-template are shown in Fig. 2, depending on their synthesis process.^44^ These colloidal-crystal-template methods rely on filling or coating the void spaces between close-packed monodisperse spheres (*e.g.*, PS, or PMMA) with liquid metal precursors and subsequent *in situ* precursor solidification. Three-dimensionally ordered macroporous (3DOM) structures can be formed after the removal of the templates by calcination at high temperature. The 3DOM structure by inverse opals are interconnected, which could favour the fast mass-transport of large molecules through the network as well as rapid gas diffusion.

Using the colloidal-crystal-template method to prepare nanoporous perovskite oxides has several advantages: (a) the ability to obtain ordered nanoporous perovskite oxides; (b) the allowance for a relatively high calcination temperature. Hur *et al.* prepared 3DOM La_0.7_Ca_0.3_MnO_3_ and La_0.7_Ca_0.32–*x*_Sr_*x*_MnO_3_ using metal alkoxide precursor solution by mixing metal acetates with stoichiometry and 2-methoxyethanol in HNO_3_, and then the viscous solution was gently dropped until the millimeter-thick PMMA template was completely soaked with the solution.^45^,^46^ Finally, the PMMA colloids were removed *via* sintering at 800 °C in an O_2_ atmosphere. However, the process to form metal alkoxide solution is time-consuming and also expensive.

Recently, a colloidal-crystal-template method using ethylene glycol–methanol solutions of various metal nitrates as precursors was developed to prepare 3DOM perovskite oxides. The ethylene glycol and metal nitrates can form a homogenous mixture, which could turn into a mixed metal glyoxylate salt following an *in situ* nitrate oxidation at a relatively low temperature before the removal of the colloidal template. Zhao *et al.* used this method to successfully obtain 3DOM LaCo_*x*_Fe_1–*x*_O_3_ perovskite oxide.^47^ Recently, some other organic surfactants and chelating agents (*e.g.*, l-lysine,^43^ poly(ethylene glycol) (PEG),^43^,^48^ F127,^48^ citric acid,^49^ glycine^50^) have been used to homogeneously disperse metal nitrates during the colloidal-crystal-template method for the synthesis of 3DOM perovskite oxides. For example, Dai *et al.* reported 3DOM La_0.6_Sr_0.4_FeO_3–*δ*_ with mesoporous or nanovoid-like skeletons using the surfactant (F127, PEG, or l-lysine)-assisted PMMA-templating method and the results show that the nature of surfactant and solvent could influence the pore structure and the surface area of the final product.^48^ In addition, the authors indicated that treating the La_0.6_Sr_0.4_FeO_3–*δ*_ precursor first in N_2_ at 500 °C to form amorphous carbon and then in air at 750 °C could favour the formation of 3DOM La_0.6_Sr_0.4_FeO_3_. Later, Dai's group used citric acid-, PEG- and P123-assisted PMMA templating methods to prepare several kinds of 3DOM perovskite oxides using a similar process, including EuFeO_3_,^51^ Eu_0.6_Sr_0.4_FeO_3_,^52^ LaMnO_3_,^43^,^53^ La_2_CuO_4_,^54^ and La_0.6_Sr_0.4_MnO_3_.^55^,^56^

More importantly, the colloidal-crystal-template method can also be used to prepare 3DOM perovskite oxides supported with noble metal nanoparticles by a one-step infiltration of the noble metal and perovskite precursor. For example, Ag nanoparticles supported on 3DOM La_0.6_Sr_0.4_MnO_3_ with high surface areas (38.2–42.7 m^2^ g^–1^) were prepared by a facile novel reduction method using PMMA as template in a dimethoxytetraethylene glycol (DMOTEG) solution, in which the DMOTEG-mediated route not only produced size controlled Ag nanoparticles, but also stabilized them against conglomeration without the need for additional stabilizers.^57^ Wang *et al.* prepared a 3DOM Pd–LaMnO_3_ composite by mixing the stoichiometric amounts of La(NO_3_)_3_·6H_2_O, Mn(NO_3_)_2_ and Pd(NO_3_)_2_·4H_2_O in methanol solution containing PEG with the l-lysine in a HNO_3_ aqueous solution.^58^

However, due to the fragile nature of the materials with wall thicknesses much thinner than the pore size, the collapse or loss of the 3D porous structure may still occur during or after the template removal.^50^ Therefore, the pore structure stability of 3DOM perovskite oxides during their preparation and applications should be carefully considered. Another drawback of the colloidal-crystal-template method is the time-consuming and expensive process for the synthesis of polymer templates, which limits the practical applications of 3DOM perovskite metal oxides. In addition, the addition of organic molecules or surfactants into the metal ion precursor solution to homogeneously disperse the metal ions is important for the formation of 3DOM perovskite oxides with a pure phase, but the possible reactions of organic molecules with colloidal-crystal templates should also be considered.

### 2.4 Electrospinning method

Electrospinning, also known as electrostatic spinning, is a technique that is used to obtain nanofibers (with diameters lower than 100 nm and lengths up to kilometres) using an electrostatically driven jet of polymer fluid stream *via* a high-voltage electric field. Electrospinning has gained substantial attention in various fields (*e.g.*, optoelectronics, sensor technology, catalysis, energy storage and conversion) due to its ability to fabricate continuous fibers with diameters down to a few nanometers, as well as various compositions (*e.g.*, polymers, polymer alloys, and polymers loaded with nanoparticles, as well as metal oxides).^59^,^60^ Over the last several years, the electrospinning method has been extended to the fabrication of perovskite oxides using homogeneous solutions containing polymer and metal nitrates as precursors. After drying, precursor nanofibers containing polymer and metal cations with stoichiometry can be obtained. After the removal of the polymer (*e.g.*, polyvinylpyrrolidone (PVP)) by calcination, perovskite oxides (*e.g.*, La_0.75_Sr_0.25_MnO_3_,^61^,^62^ LaCoO_3_,^63^,^64^ LaFeO_3_,^65^,^66^ BiFeO_3_,^67^ La_0.5_Sr_0.5_CoO_3–*x*_,^68^ La_0.5_Sr_0.5_Co_0.8_Fe_0.2_O_3_,^69^ La_0.6_Sr_0.4_Co_1–*x*_Fe_*x*_O_3–*δ*_ (ref. 70)) with pure phase can be obtained. If the composition and calcination process is carefully controlled, nanoporous perovskite oxides can be formed by the electrospinning method.

The addition of organic molecules (*e.g.*, citric acid) into the precursor for electrospinning is also important for the synthesis of nanoporous perovskite oxides with pure phase because of their ability to maintain a good dispersion of the perovskite oxide precursors and also provide heat for the formation of crystals, similar to that of soft-template, hard-template and colloidal-crystal-template methods. For instance, Song *et al.* prepared perovskite LaCoO_3_, LaMnO_3_, LaFeO_3_, La_0.8_Sr_0.2_CoO_3–*δ*_ and La_0.9_Ce_0.1_CoO_3_ nanofibers with 3D porous structures and high surface areas (27–60 m^2^ g^–1^) using the electrospinning method combined with heat treatment at 350 °C, in which metal nitrates, citric acid and PVP dissolved in H_2_O was used as precursor solution.^66^ In Lin's paper about the synthesis of PrFeO_3_ porous nanotubes by electrospinning, citric acid and PVP were also used in the precursor solution and they found that the evaporation of the residual solvent and moisture at 40 °C induced the higher concentration of metal nitrate and citric acid in the surface layer of fibers than that in the core area, leading to the formation of a porous PrFeO_3_ sheath layer due to the thermal decomposition of metal nitrates and citric acid and the structural collapse in the core through calcination.^71^ The results show that the annealing treatment of the fibers would be helpful for the formation of nanoporous structure but the detailed reasons still need more research.

### 2.5 Hydrothermal method

The hydrothermal method has been widely used to prepare nanocrystals due to its several advantages, such as high yield, easy control, low air pollution, low energy consumption, ability to create crystalline phases with good quality which are not stable at the melting point and so on.^72^ In recent years, the hydrothermal method has also been used for the synthesis of nanoporous perovskite oxides, however, the most widely investigated nanoporous perovskite oxides *via* hydrothermal method are mainly based on titanates, such as SrTiO_3_,^73^ BaTiO_3_,^74^ PbTiO_3_.^75^

**Fig. 3:**
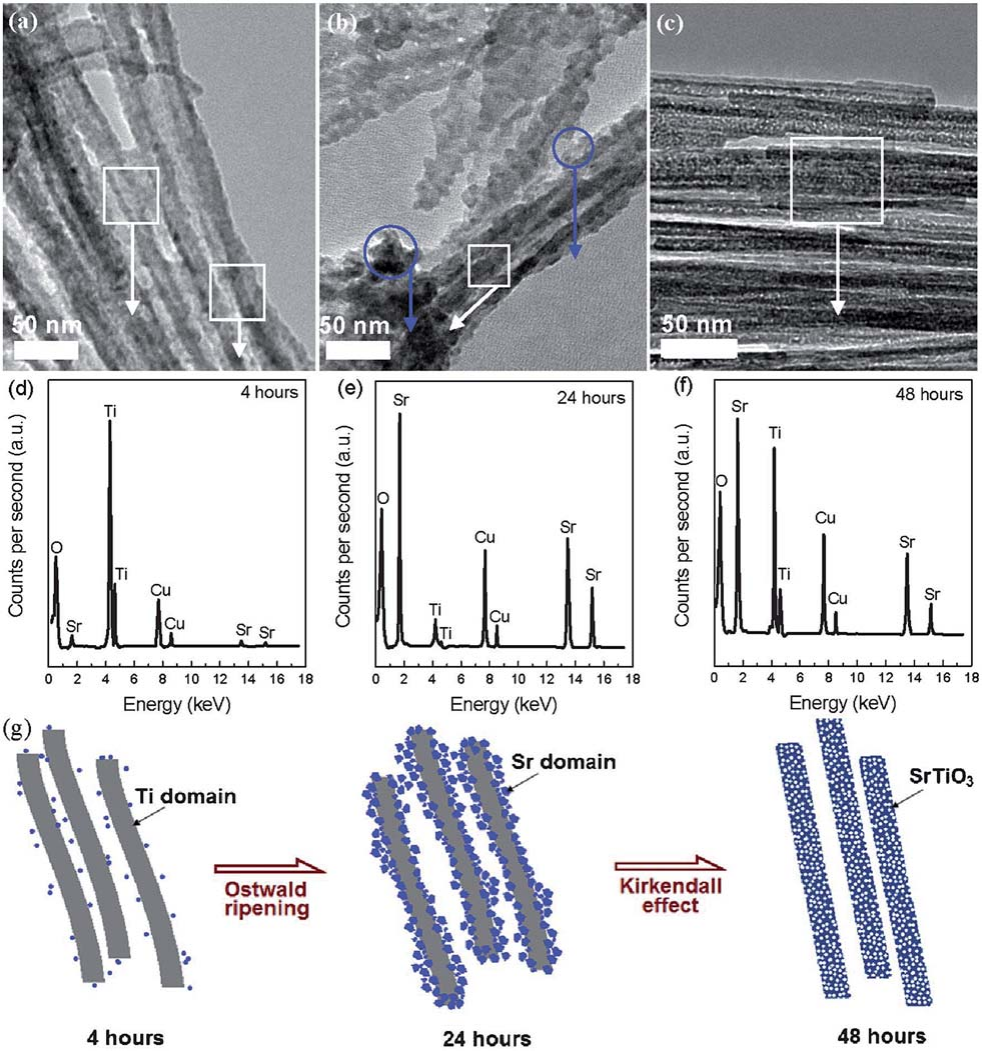
TEM (a–c) and EDS (d–f) images for aliquots of the mixture after autoclaving for different time. Regions where EDS spectra were taken are circled in the TEM images. A proposed mechanism for the mesoporous nanowires (g). Reprinted with permission from ref. 73. Copyright© 2012, Royal Society of Chemistry.

For the nanoporosity design of titanate-based perovskite oxides (*e.g.*, SrTiO_3_) *via* hydrothermal method, one strategy is to use TiO_2_ or layered protonated titanate as a template without any soft templates. Recently, Wang *et al.* prepared porous perovskite titanates (*i.e.*, SrTiO_3_, BaTiO_3_, and CaTiO_3_) *via* a hydrothermal processes, in which amorphous TiO_2_ spheres with different textural properties (*i.e.*, size, porosity, and structure) were used as template for the hydrothermal reaction with alkaline earth metal ions.^76^ They indicate that the underlying formation mechanism includes *in situ* crystallization or Ostwald ripening during the hydrothermal process.^76^ Nanoporous perovskite oxides can also be prepared *via* combined sol–gel and hydrothermal method. Ren *et al.* reported mesoporous SrTiO_3_ nanowires *via* a one-step template-free hydrothermal method, in which tetrabutyl titanate in propanol was added into a Sr(NO_3_)_2_ aqueous solution under gentle stirring and the pH value was adjusted to 13 by NaOH. After hydrothermal reaction at 160 °C for 48 h and calcination at 500 °C for 6 h, mesoporous SrTiO_3_ nanowires with surface areas of 145 m^2^ g^–1^ and the pore volume of 0.43 cm^3^ g^–1^ can be obtained.^73^ The authors suggested that the growth and morphology evolution mechanism of the well-structured mesoporous SrTiO_3_ nanowires were governed by the Ostwald ripening process combined with the Kirkendall effect, as shown in Fig. 3.^73^

In addition, polymer templates (*e.g.*, poly(vinyl alcohol), CTAB, poly(vinyl chloride)-*g*-poly(oxyethylene methacrylate)) and other additives (*e.g.*, Na_2_SiO_3_·9H_2_O) as soft or hard templates were also reported for the fabrication of nanoporous perovskite oxides based on titanates (*e.g.*, SrTiO_3_ (ref. 77)) *via* a hydrothermal route. These polymer templates or additives can provide templates for pore formation during the calcination process after the hydrothermal reaction.

Besides the titanate-based perovskite oxides, some other nanoporous perovskite oxides (*e.g.*, LaFeO_3_,^78^ LaNiO_3_ (ref. 79)) can also be prepared *via* the hydrothermal method. For example, Dai *et al.* reported a glucose-assisted hydrothermal method to prepare nanoporous LaFeO_3_ samples with surface areas of 15–26 m^2^ g^–1^.^78^ They investigated the effect of hydrothermal temperature on the morphology, surface area, pore structure, surface oxygen concentration and found that the sample obtained at 170 °C showed the highest surface area (26 m^2^ g^–1^) and the highest surface oxygen concentration.

### 2.6 Summary of preparation methods

**Fig. 4:**
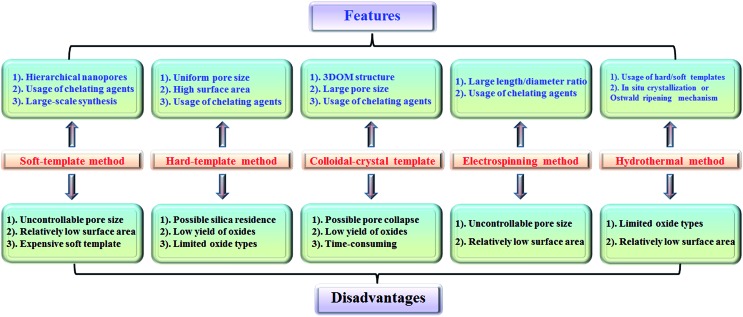
Summary of synthesis methods for nanoporous perovskite metal oxides.

The features and disadvantages of various synthesis methods of nanoporous perovskite oxides are summarized and prospected in Fig. 4. The synthesis of nanoporous perovskite oxides *via* soft-template method is relatively easy and can be carried out under mild conditions on a large scale. In addition, a variety of hierarchically nanoporous perovskite oxides can be prepared depending on the type of soft template, the composition, and also organic additives. However, it is difficult to exactly control the nanoporous structures due to the complex sol–gel processes and the polymerization of metal cations, as well as the poor thermal stability. The hard-template method offers some advantages, such as controlled nanostructures by choosing hard templates with desired structures, ability to obtain uniform nanoporous perovskite oxides with highly crystalline walls and high surface area. However, it is usually difficult to incorporate enough metal precursors into the nanopores of the hard-template at one time, which would lead to the formation of perovskite oxides in bulk. In addition, the targeted nanoporous perovskite oxides must be stable in NaOH or HF solutions which are used to remove the mesoporous silica templates. Furthermore, the possible silica residences left after NaOH or HF treatment may contaminate the final products and affect the properties of nanoporous perovskite oxides. Even though mesoporous carbon used as hard templates can be completely removed by calcination, the poor wetting of the pore walls by the aqueous precursor solution limits its wide applications in the synthesis of nanoporous perovskite oxides.

The colloidal-crystal-template method provides an easy and controlled way to prepare 3DOM perovskite oxides *via* the complete removal of polymer microspheres (*e.g.*, PS, PMMA), however the poor thermal stability of 3DOM perovskite oxides also exists due to the thin wall compared with the macropores, but the synthesis of polymer microspheres with controlled properties in most cases is complex and time-consuming.

Even though the electrospinning and hydrothermal methods have been reported for the preparation of nanoporous perovskite oxides, the perovskite oxide compositions are limited to certain types. The hydrothermal method is mainly used for the synthesis of nanoporous perovskite oxides based on titanates, while in the electrospinning method it is difficult to control the pore structure and increase the surface area due to the high calcination temperature and small portion of metal precursor. In summary, each synthesis method has its advantages, disadvantages, as well as limitations. Among these methods, soft-template and colloidal-crystal-template methods are relatively easier to operate and scale up. In addition, the polymer template can be completely removed by calcination and there is no template residue left in the obtained nanoporous perovskite oxides, which is an important property for special applications. Novel techniques or strategies based on soft-template or colloidal-crystal-template methods still need to be developed to obtain more uniform or stable nanoporous structures in a facile route.

### 2.7 Special points in synthesis of nanoporous perovskite oxides

All these synthesis methods mentioned above have also been applied for the synthesis of nanoporous single metal oxides. However, there are some special points in the synthesis of nanoporous perovskite oxides mainly due to their high temperature for crystallization and complex multi-metal ions composition. The high calcination temperatures for the formation of pure-phased perovskite oxides usually lead to poor thermal stability of the nanoporous structure, especially when using the soft-template and electrospinning methods. In addition, the multi-metal cations should be homogeneously dispersed during the whole process with the purpose of obtaining pure-phase perovskite oxides. As a result, small organic chelating agents, such as citric acid and acetic acid, are typically used in the synthesis of nanoporous perovskite oxides associated with the soft template, hard template, or colloidal crystal template. The large amount of heat released from the decomposition of organic molecules is also favourable to the formation of pure-phased perovskite oxides at relatively low temperatures.

## 3. Applications

Bulk perovskite oxides have been widely used in catalysis, energy storage and conversion, *etc.*^80^ In addition, as demonstrated in many nanoporous materials (*e.g.*, mesoporous silica, nanoporous metal oxides, mesoporous carbons), the surface areas and particle size greatly affect their properties and performance.^81^ It is possible that nanoporous perovskite oxides with high surface areas could allow the adsorption of reactants on both the outside surface and inside the pores, maybe further improving their performance. Herein, the applications of nanoporous perovskite oxides as catalysts for chemical catalysis (*e.g.*, methane combustion, toluene oxidation, soot oxidation), electrochemical catalysis (*e.g.*, cathodes for SOFCs or metal–air batteries), photocatalysis (*e.g.*, photocatalytic degradation, photocatalytic water splitting and CO_2_ reduction), *etc.* will be summarized and highlighted.

### 3.1 Chemical catalysis

Perovskite oxides have potential catalytic applications either for high-temperature gas or solid reactions, or liquid reactions at low temperatures because of their high thermal and hydrothermal stability, good redox ability, excellent oxygen mobility, as well as a wide range of stoichiometries and crystal structures.^82^,^83^ Among the parameters affecting the catalytic activity, the textural property plays a critical role due to its direct determination to active sites accessible for reactants. Nanoporous perovskite oxides with higher surface areas and more catalytically active sites might exhibit improved catalytic performance as compared with their bulk counterparts.

#### 3.1.1 Gas combustion

Catalytic combustion of fuels and volatile organic compounds (VOCs), such as methane and toluene, is important for power generation and environmental protection. Nanoporous perovskite oxides have been widely used as catalytic materials for the abatement of exhaust gases, such as methane and toluene. For example, Lu *et al.* reported a three-dimensional ordered mesoporous LaCoO_3_ perovskite oxide with high surface area (*i.e.*, 96.7 m^2^ g^–1^) *via* the nanocasting strategy by using ordered mesoporous cubic (*Ia*3*d*) vinyl silica as the template.^36^ The mesoporous LaCoO_3_ perovskite showed much higher performance in the methane combustion than the bulk LaCoO_3_ prepared by a conventional citrate method. The H_2_-TPR and X-ray photoelectron spectroscopy results show that the high valent cobalt ions and high content of O_2_^2–^/O^–^ species in the mesoporous LaCoO_3_ sample resulted in higher catalytic activity, indicating high surface areas would be favorable for the formation of high valent metal ions and high content of active oxygen species.

In addition, 3DOM perovskite oxides (*e.g.*, La_0.6_Sr_0.4_MnO_3_,^55^ La_2_CuO_4_ (ref. 54)) or their supported metal nanoparticle composites (*e.g.*, Ag/La_0.6_Sr_0.4_MnO_3_,^56^,^57^ Pd–LaMnO_3_ (ref. 58)) prepared by colloidal-crystal-template method have also been investigated as catalysts for methane combustion and demonstrated to show high catalytic activity. Dai *et al.* compared the catalytic performances of Ag/3DOM La_0.6_Sr_0.4_MnO_3_ composites with different Ag loading amounts for methane combustion, demonstrating that 3.63 wt% Ag/3DOM La_0.6_Sr_0.4_MnO_3_ shows super catalytic activity, which contributed to its larger surface area, higher oxygen adspecies concentration, better low-temperature reducibility, and unique nanovoid 3DOM structure.^56^

Besides the applications in the catalytic combustion of methane, nanoporous perovskite oxides have been applied as catalysts for catalytic combustion of VOCs (*e.g.*, CO, toluene) over the years due to their stable structure, and especially, excellent catalytic performances.^84^,^85^ Dai *et al.* investigated the activity of ErFeO_3_-3DOM and Er_0.6_Sr_0.4_FeO_3_-3DOM catalysts with a surface area of 16–31 m^2^ g^–1^ on the combustion of toluene and the order in catalytic activity sequence decreased in terms of Er_0.6_Sr_0.4_FeO_3_-3DOM > ErFeO_3_-3DOM > ErFeO_3_-bulk, in good agreement with surface oxygen species concentration, increased valence of transition metal and low-temperature reducibility, which is directly related with the nanoporous structure.^49^

#### 3.1.2 Soot oxidation

Diesel soot is another typical pollutant which needs to be oxidized avoiding emission into the atmosphere for environmental clean-up. The solid state of soot limits its free diffusion and spread on the catalyst's surface, as a result, the catalytic oxidation of soot is usually complex and requires multifunctional catalysts with excellent activity. The theory of the oxidation of solid soot by oxygen, steam, carbon dioxide or nitrogen dioxide under catalyst surface involves the adsorption of gaseous oxidizers, dissociative adsorption of oxygen onto the catalyst surface, and soot oxidation by the active oxygen species.^86^ Therefore, the tight contact of soot and catalyst, the rapid diffusion of oxidant and gaseous products are of crucial importance for the excellent catalytic performance.

Recently, nanoporous perovskite oxides have been applied as catalysts for soot oxidation.^87^,^88^ For example, Zhao *et al.* found that the 3DOM LaCo_0.5_Fe_0.5_O_3_ catalyst had better catalytic performances than the non-macroporous particle catalysts for diesel soot combustion because the 3DOM perovskite catalysts with higher surface areas and ratio of surface to bulk atoms could favor the contact between the catalyst and the reactants (soot and oxygen), resulting in a decreased ignition temperature and improved catalytic performances over non-macroporous particles.^47^ Later, the authors found that the pore diameters of 3DOM LaMn_1–*x*_Fe_*x*_O_3_ catalysts also affected greatly their catalytic activity, in which the 3DOM LaMn_1–*x*_Fe_*x*_O_3_ catalyst with pore diameter above 400 nm had the highest catalytic activity for soot combustion due to its better contact with the soot. In addition, the authors found that the synergistic effect between the deposited Au nanoparticles with *ca.* 3 nm and the 3DOM LaFeO_3_ perovskite oxides could lead to a further decreased ignition temperature for soot oxidation.^89^

#### 3.1.3 Other chemical catalysis

Besides the abovementioned reactions, nanoporous perovskite oxides have also been investigated as promising catalysts for various other reactions, such as methyl chloride oxidation,^32^ methane dry reforming with CO_2_,^33^ methanol oxidation,^37^ oxidative degradation of organic dyes,^90^ reduction of NO,^38^ liquid phase Friedel–Crafts benzylation reactions,^91^ and chemical-looping steam reforming of methane.^92^ More information about these applications can be seen from the referred literature.

### 3.2 Electrochemical catalysis

Today, energy storage and conversion are of great importance to the sustainable world. Perovskite oxides have been widely investigated as electrochemical oxygen reduction reaction (ORR) catalysts for potential applications in energy storage and conversion fields, especially in solid oxide fuel cells (SOFCs) and metal–air batteries.^93^–^95^ The A-site and/or B-site substitutions and crystalline structure all affect their performance as ORR catalysts. In addition, their morphology has a great influence on their performance.

#### 3.2.1 Solid oxide fuel cells

SOFCs have attracted increasing interest because of its cleanness, high efficiency, and versatility for chemical-to-electrical energy conversion.^96^,^97^ However, the high-operating temperatures (*e.g.*, 800–1000 °C) of commercial SOFCs bring in strict requirements to the electrode and interconnected materials. Therefore, much effort has been devoted to the development of low-temperature SOFCs, which is believed to greatly reduce the cost of materials required for cell fabrication, at the same time improving reliability and operational life.^96^ However, the reduced operating temperature would result in increased interfacial polarization resistances between electrodes and electrolyte. Traditionally, the development of novel electrode materials exhibiting outstanding properties is a conventional route to improve the performance and durability of SOFCs. Recently, integration of unique micro- and nanostructured materials may be one of the most promising strategies in the commercial applications of SOFCs.^98^,^99^ More discussion about the nature and requirements of SOFCs can be seen in other reviews.^97^,^100^ Perovskite oxides with high ionic/electronic conductivity have been widely applied as anode, cathode and electrolyte in SOFCs. Among these, the microstructure of cathode materials is considered the most possible to be tuned in SOFCs.

To date, multiscaled nanoporous mixed ionic/electronic conductors (MIECs) are attractive electrodes for intermediate- or low-temperature SOFCs because the usage of nanoporous MIECs can extend the active electrochemical sites from the triple-phase boundaries (TPBs) at the interface between an electronic conductor electrode and the electrolyte. In addition, the pore architecture of the nanoporous electrode also has great influence on the gas transport and the catalytic activity of the interfaces. There are a number of nanoporous electrode materials for SOFCs reported, including La_1.7_Ca_0.3_Ni_0.75_Cu_0.25_O_4–*δ*_,^16^ La_0.6_Sr_0.4_Co_0.2_Fe_0.8_O_3–*δ*_,^101^ and Sm_0.5_Sr_0.5_Co_3–*δ*_.^102^

Infiltration of active components into a porous electrode scaffold is a facile method to improve the electrochemical performance of cathodes for low-temperature SOFCs. The infiltration method also provides a facile strategy to prepare nanoporous cathode materials for SOFCs. For example, Chen *et al.* used a self-rising approach using P123 and urea as pore former to prepare infiltrated multiscale nanoporous Sm_0.5_Sr_0.5_CoO_3–*δ*_–BaZr_0.1_Ce_0.7_Y_0.2_O_3_ composite cathode for proton-conducting SOFCs, in which the gases released from the decomposition of urea at elevated temperatures can be used as a macropore generator.^21^ Polarization resistance of the cell consisting of multiscaled nanoporous Sm_0.5_Sr_0.5_CoO_3–*δ*_–BaZr_0.1_Ce_0.7_Y_0.2_O_3_ composite was 0.388 ohm cm^2^ at 600 °C while screen-printed Sm_0.5_Sr_0.5_CoO_3–*δ*_–BaZr_0.1_Ce_0.7_Y_0.2_O_3_ composite cathode showed a much higher polarization of 0.912 ohm cm^2^ at 600 °C. Recently, Chen's group developed a vacuum-free infiltration and subsequent freeze-drying combustion method to prepare hierarchically nanoporous electrode materials, including cathode network Sm_0.5_Sr_0.5_CoO_3–*δ*_–Gd_0.1_Ce_0.9_O_1.95_ (SSC–GDC) and anode Ni–GDC.^102^,^103^ The results show that both the activation polarization loss and gas diffusion resistance could be decreased due to the novel electrodes microstructure. The performance of fuel cells using the porous SSC–GDC cathode is much better than the others due to the significantly facilitated mass transport, extended effective TPB length and increased reaction sites, and accelerated oxygen reduction process.

**Fig. 5:**
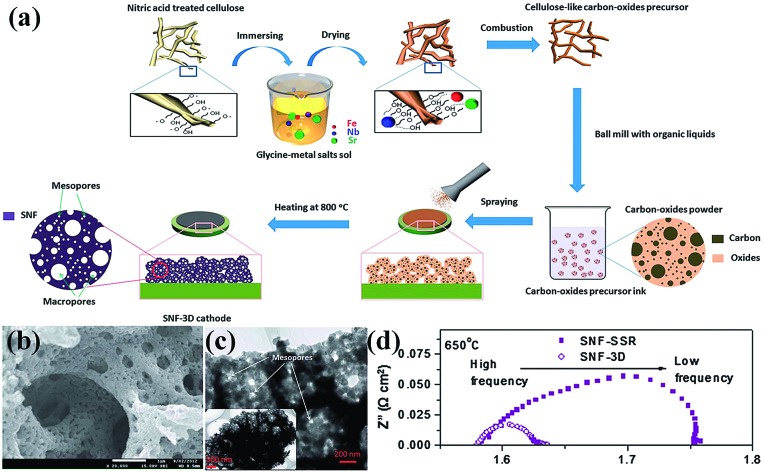
(a) Procedure for the preparation, (b) SEM and (c) TEM images of a 3D aperiodic hierarchical porous SNF cathode calcined at 800 °C for 2 h in air, and (d) electrochemical impedance spectroscopy of SNF-3D and SNF-SSR (SSR: solid state reaction) at 650 °C. Reproduced with permission from ref. 104. Copyright© 2012, Royal Society of Chemistry.

Nanoporous perovskite cathode materials can also be firstly prepared *via* various methods and then coated onto the surface of electrolytes by sintering or spraying at high temperature. The nanoporous structure of cathode materials should be stable during the sintering process. Huang *et al.* reported a citrate-modified EISA method using P123 and citric acid as surfactants to prepare hierarchically nanoporous La_1.7_Ca_0.3_Ni_1–*x*_Cu_*x*_O_4–*δ*_ layered perovskite oxides and investigated as cathodes for Y_2_O_3_-stabilized ZrO_2_ (YSZ) anode-supported IT-SOFCs.^16^ The results show that the hierarchically nanoporous La_1.7_Ca_0.3_Ni_0.75_Cu_0.25_O_4–*δ*_ cathode exhibited higher electrochemical performance and better adhesion ability with YSZ electrolyte than their bulk La_1.7_Ca_0.3_Ni_0.75_Cu_0.25_O_4–*δ*_ counterparts prepared *via* a conventional citrate method and a solid state reaction. Shao *et al.* reported that the oxygen surface processes, gas transport and electrochemical performance (*e.g.*, power generation ability) were significantly improved in 3D SrNb_0.1_Fe_0.9_O_3–*δ*_ (SNF-3D) cathode fabricated directly from a carbon-oxides precursor due to the highly improved surface areas and the interconnected hierarchical pores, as shown in Fig. 5.^104^

Perovskite electrode materials prepared by a colloidal-crystal-template method with PMMA spheres have also been investigated in SOFCs field.^50^ For example, Nunez *et al.* used PMMA microspheres as pore formers to prepare thin layer oxide materials with a controlled macroporous microstructure.^105^ They compared the electrochemical performance of LSM–YSZ, Ba_0.5_Sr_0.5_Co_0.8_Fe_0.2_O_3–*δ*_–YSZ cathode and NiO–YSZ as anode with and without microstructural optimisation and microstructural optimisation using PMMA could achieve a higher performance.^106^

Fundamental research focused on the nanoporous structure effects on the electrochemical performance for perovskite oxides in SOFCs has been widely investigated in the past few years. It turns out that the nanoporous perovskite oxides exhibit higher powder density and lower area specific polarization, as compared with their counterparts without nanoporous structures. Optimizing the nanoporous structure of MIEC cathode materials is one of the best approaches for lowering the operating temperature for SOFCs whilst retaining high electrochemical performance.^107^

#### 3.2.2 Electrodes for low temperature fuel cells and metal–air batteries

Metal–air batteries have attracted considerable attention in the last decade due to their much higher theoretical power densities than most currently available primary and rechargeable batteries. Although the theoretical energy densities of metal–air batteries are high, for example 11680 W h kg^–1^ for Li–air battery, the realization of their commercialization still faces many challenges, including low practical energy density and efficiency, poor rate capacity, and short cycle life. Therefore, it is of crucial importance to develop highly efficient electrocatalysts for metal–air batteries. Perovskite oxides have a high electronic/ionic conductivity and catalytic activity, and thus could be promising candidates as ORR or OER catalysts in fuel cells and metal–air batteries. Various kinds of perovskite oxides with different compositions, morphologies and phase structures have been reported with applications in metal–air batteries, including LaFeO_3_,^41^ LaCoO_3_,^63^ LaNiO_3_,^79^,^108^ CaMnO_3_,^109^ Sm_0.5_Sr_0.5_CoO_3_,^110^ LaNi_0.9_M_0.1_O_3_ (M = Cu, Co).^111^

Nanoporous perovskite oxides with high surface areas and pore structures may enable electron and oxygen migration and could also stabilize specific oxygenated intermediates to boost catalysis. Recently, Huang *et al.* reported that nanoporous La_0.6_Ca_0.4_CoO_3_ with a specific surface area of over 210 m^2^ g^–1^ exhibits higher electrocatalytic activity for bi-functional catalysis in an alkaline electrolyte than particle nanostructured La_0.6_Ca_0.4_CoO_3_ mainly due to the high surface area and the novel nanostructure.^112^ Mai *et al.* demonstrated the high performance of hierarchically mesoporous La_0.5_Sr_0.5_CoO_2.91_ nanowires for ORR with low peak-up potential and high limiting diffusion current, and ultrahigh capacity (*ca.* over 11 000 mA h g^–1^) in nonaqueous Li-air batteries, one order of magnitude higher than that of La_0.5_Sr_0.5_CoO_2.91_ nanoparticles.^113^ Similarly, nanoporous La_0.75_Sr_0.25_MnO_3_ nanotubes also exhibited better performance (*e.g.*, improved round-trip efficiency, higher specific capacity, superior rate capability and better cycle stability) than Ketjenblack carbon (KB) carbon and nonporous La_0.75_Sr_0.25_MnO_3_ for rechargeable lithium–O_2_ batteries due to the fast electron transport, short diffusion distances for O_2_ and electrolyte, and large electrode–electrolyte contact area to ensure high availability of the catalytic active sites.^62^

### 3.3 Photocatalysis

Photocatalysis has been studied for a long time since the discovery of photocatalytic activity in TiO_2_ in 1970s.^114^ A photocatalytic reaction primarily involves the following processes: the generation of electron–hole pairs by photocatalysts due to the photoexcitation, then the subsequent charge separation and migration to the surface and finally the reduction/oxidation reaction on the surface of photocatalysts.^115^–^117^ The number of materials systems and applications based on photocatalysis has increased sharply in the last several decades, in which perovskite oxides are one of the most important families of materials suitable for photocatalysis due to their stability, flexible component and structure engineering.^118^,^119^

#### 3.3.1 Photocatalytic degradation

Considerable information is available on photocatalytic degradation, in terms of reactions, mechanism, as well as performance of various photocatalysts.^120^,^121^ Perovskite-type semiconductors have shown excellent photocatalytic properties due to several significant advantages over the corresponding binary oxides, such as favourable band edge potentials, broader scopes to tune band structure as well as other photophysical properties, and the ability to combine with effects such as ferroelectricity or piezoelectricity.^118^,^122^

SrTiO_3_, a typical perovskite-type oxide with a band gap of 3.2 eV, has been widely investigated as photocatalysts owing to its extraordinary physicochemical properties such as excellent thermal stability and photocorrosion resistibility. Nanoporous SrTiO_3_ structures with high crystallinity will potentially enhance its photocatalytic performance due to the improved surface exchange from unique porous structure and large surface-to-volume ratio. In recent years, several types of nanoporous structured SrTiO_3_ have been reported as photocatalysts in photocatalytic degradation. For example, Zou *et al.* reported mesoporous perovskite oxides SrTiO_3_ or BaTiO_3_ as photocatalyst for photocatalytic dehydrogenation of 2-propanol to acetone under light irradiation (*λ* > 300 nm) and the mesoporous SrTiO_3_ (*e.g.*, MST-1.5 and MST-2) and BaTiO_3_ (*e.g.*, MBT-1.5) both exhibited much higher activities than those of the samples prepared by the solid-state reaction, which can be attributed to the larger specific surface areas and active sites for the catalytic reaction.^123^

In addition, mesoporous doped SrTiO_3_ oxides have also been reported to show excellent photocatalytic degradation activity due to its good structural stability. Sreethawong, *et al.* reported mesoporous-assembled SrTi_*x*_Zr_1–*x*_O_3_-based nanocrystals photocatalysts for azo dye degradation.^124^ The results show that without Pt loading, the mesoporous-assembled SrTi_0.9_Zr_0.1_O_3_ photocatalyst calcined at 700 °C for 4 h provided a maximum degradation rate constant as compared to the other SrTi_*x*_Zr_1–*x*_O_3_ photocatalysts. Besides the large surface area and developed porosity in mesoporous doped SrTiO_3_, the N-doping also contributed to its excellent activity for photodegradation of organic dyes under visible-light (*λ* > 420 nm).^125^

Besides nanoporous perovskite oxides based on titanate (*e.g.*, SrTiO_3_, BaTiO_3_, ZnTiO_3_), some other types of nanoporous perovskite oxides based on ferrite (*e.g.*, PrFeO_3_,^71^ BiFeO_3_,^19^,^126^–^128^) have also been reported as photocatalysts in degradation. For example, mesoporous BiFeO_3_ nanoarchitectures with a direct optical band gap at 2.9 eV exhibited a much better catalytic activity for the degradation of RhB than that of nontemplated samples, which can be attributed to the nanoscale porosity with more available active sites and favourable reactant adsorption and diffusion.^19^

Although a large number of perovskite-based oxides have been reported for photocatalytic degradation, the reports about the nanoporous perovskite oxides as photocatalysts on degradation are still limited, especially under visible light. In addition, SrTiO_3_-based nanoporous photocatalysts are the mostly investigated systems. Even though several reports have demonstrated the beneficial effect of nanoporous morphology on the photocatalytic activity, more efforts are needed to understand the structure–morphology–property relations in nanoporous perovskite oxides and their photocatalytic activity. In addition, multiple doping and combining the nanoporous morphology in the perovskite oxides should be effective strategies to enhance photocatalytic activity under visible light. Nonetheless, very limited knowledge is available on the possible adverse effects of dopants and nanoporous morphology on the photocatalytic activities of these compounds. This needs to be carefully investigated in the future.

#### 3.3.2 Photocatalytic water splitting and CO_2_ reduction

Photocatalytic water splitting for H_2_ generation and CO_2_ reduction into chemical fuels by perovskite oxides has also been reported.^129^–^132^ Even though the photon energy conversion and water splitting efficiency of the reported photocatalysts is still not at the stage of practical application, research in photocatalytic water splitting is being advanced, with several reviews about photocatalytic water splitting have been published.^115^,^116^,^130^,^133^ Photocatalytic water splitting is more difficult and distinguished than photocatalytic degradation, therefore sacrificial reagents (*e.g.*, alcohol as electron donors or Ag^+^ as hole scavengers) are often employed to evaluate whether a certain photocatalyst could satisfy the thermodynamic and kinetic potentials for H_2_ and O_2_ evolution.

The nanoporous perovskite photocatalysts could favour the interaction of reactants with photocatalytic active sites, the separation of electrons and holes, as well as the H_2_ diffusion, resulting in higher photocatalytic efficiency. For example, mesoporous SrTiO_3_ photocatalysts exhibited much higher hydrogen production from photocatalytic water splitting using methanol as the hole scavenger than both non-mesoporous-assembled commercial SrTiO_3_ and commercial TiO_2_ (P25).^134^ Similar results have also been observed by Zou,^135^ who reported a high rate of photocatalytic H_2_ evolution by nanoporous SrTiO_3_ nanocrystals with a cellular morphology as efficient photocatalytic hydrogen production, which is attributed to their large specific surface area and high crystallinity. The smaller particle size, larger surface area and lower Ti^3+^ amount of nanoporous Sr-rich SrTiO_3_ (denoted as SrTiO_3_-1) could also lead to a higher photo-generated charge separation efficiency and a H_2_ evolution rate more than three times as high as that of non-porous SrTiO_3_ sample (SrTiO_3_-2), demonstrated by Li *et al.*^136^

For photocatalytic CO_2_ reduction, several factors should be considered, including CO_2_ capture (CO_2_ diffusion/adsorption), light harvesting, gas conversion (multi-electron chemical reactions) and electron–hole separation. The nanoporous structures with high specific surface areas and active sites have been demonstrated by several researchers to significantly enhance the CO_2_ photocatalytic reduction efficiency.^137^–^139^ Some nanoporous perovskite photocatalysts have been reported as photocatalysts for CO_2_ reduction into chemical fuels. For example, Ye *et al.* reported a leaf-shaped 3D hierarchical artificial photosynthetic system of perovskite ATiO_3_ (A = Sr, Ca, and Pb) towards CO_2_ photoreduction into hydrocarbon fuels (CO and CH_4_) using water as an electron donor under artificial sunlight, in which the 3D architecture was performed as an efficient mass flow network for improved gas diffusion and light harvesting.^140^ Recently, Ye *et al.* reported photocatalytic reduction of CO_2_ into CO and hydrocarbons by hydrous hydrazine over Au–Cu bimetallic alloy nanoparticles supported on SrTiO_3_/TiO_2_ coaxial nanotube arrays, with the synergetic catalytic effect between the Au–Cu alloy nanoparticles and the fast electron-transfer in SrTiO_3_/TiO_2_ coaxial nanoarchitecture being the main reasons for the efficiency.^141^

Nanoporous perovskite oxides could exhibit high photocatalytic activity and stability due to their large surface areas, abundant active sites, quick interfacial transport, and high utilization of light arising from multi reflections in the pores.^142^ In addition, other features, such as high crystallinity, low defect density, short charge-transfer distance, and special morphology are also important for enhanced photocatalytic performance. As summarized, the photocatalytic water splitting and CO_2_ reduction are still a challenging reaction and there is a long way to go. There are not many reports about the applications of nanoporous perovskite oxides as water splitting and CO_2_ reduction photocatalysts. More efficient nanoporous perovskite photocatalysts with regards to the composition, pore size and morphology should be developed for water splitting and CO_2_ photocatalytic reduction.

### 3.4 Other fields

Due to the important properties of perovskite oxides, nanoporous perovskite oxides have also been studied for various other applications. Nanoporous perovskite oxides have also been studied in dye-sensitized solar cells using SrTiO_3_, CaTiO_3_ and BaTiO_3_ nanoporous film electrodes,^143^ or mesoporous BaSnO_3_ layer,^144^ gas sensor based on nanoporous LaCoO_3_,^64^ LaFeO_3_ (ref. 145–147) or La_1–*x*_Mg_*x*_FeO_3_,^148^ magnetoresistance and electrostatic control of magnetism in ordered mesoporous La_1–*x*_Ca_*x*_MnO_3_,^22^ ferroelectricity using mesoporous BaTiO_3_ and SrTiO_3_,^24^,^26^ and H_2_O_2_ reduction using nanoporous La_1–*x*_Ca_*x*_CoO_3_.^149^,^150^

### 3.5 Advantages and challenges of nanoporous perovskite oxides in applications

Perovskite oxides are renowned for their good stability; however nanoscale geometry does facilitate chemical attack and other degradation phenomena through increased surface area. Increased order as in nanoporous somewhat mitigates stability concerns, especially where the nanoporous structure exhibits good crystallinity.

In the catalysis field, the contact of reactants and catalyst, the diffusion of reactants and products, and the amount of surface active sites, *etc.* are important factors for the catalytic performance. Nanoporous perovskite oxides could exhibit better catalytic performance as compared with their nonporous and bulk counterparts because of the higher surface areas, more catalytically active sites, higher content of active species (*e.g.*, O_2_^2–^/O^–^, high valence metal ions), as well as rapid mass diffusion of nanoporous perovskite oxides in chemical catalysis field.

For electrochemical catalysis, the nanoporous perovskite oxides also show enhanced electrochemical performance (*e.g.*, decreased activation polarization loss and gas diffusion resistance) due to the significantly facilitated gas transport, extended effective TPB length, increased reaction sites, accelerated oxygen reduction process. Besides the nanoporous structure construction, the ionic/electronic conductivity of perovskite oxides should also be high enough for the rapid transport of electrons and ions.

Even though nanoporous perovskite oxides have also been demonstrated to exhibit high photocatalytic activity due to their high surface areas, abundant active sites, and high utilization of light arising from multi reflections in the pores, low crystallinity and high surface defect density are also usually existed in nanoporous perovskite oxides, which are usually not favourable for the photo-generated electron–hole pair separation. Therefore, other factors, such as the crystallinity, element-doping and band gap engineering of perovskite-oxide based photocatalysts, should also be considered in their photocatalytic activity.

Indeed, special concern on nanoporous structure stability (*e.g.*, morphology and chemical stability) during preparation and applications, ionic/electronic conductivity, element doping, and surface modification of perovskite oxides should be paid to enhance their performance across the full range of applications.

## 4. Conclusions and outlook

Nanoporous perovskite oxides with high surface areas and unique physicochemical properties have a wide range of potential applications. In recent years, a variety of nanoporous perovskite oxides have been synthesized by several different kinds of method (*e.g.*, soft-, hard- and colloidal-crystal-template methods, electrospinning method, and hydrothermal method), and their unique properties and applications depending on porous morphology have been investigated. Here we have given a brief survey of the methods for synthesizing nanoporous perovskite oxides, and their applications in three main fields (*e.g.*, chemical catalysis, electrochemical catalysis and photocatalysis). Each synthesis method has its own advantages and disadvantages. The hard-template method can be used to prepare ordered nanoporous perovskite oxides, but the preparation is expensive, time-consuming and possible silica residual contamination. Even it is difficult to prepare ordered nanoporous perovskite oxides using the soft template, it can obtain hierarchically nanoporous perovskite oxides with pure phase at a large scale. The colloidal-crystal-template method can be used to obtain 3DOM perovskite oxides with pure phase even though the synthesis of PMMA microspheres with desired size is also time-consuming and difficult to control. Among these synthesis methods, the addition of organic chelating agents (*e.g.*, acetic acid, citric acid) besides the pore formation surfactants or templates (*e.g.*, PMMA microspheres, P123, F127) into the precursors is important for the formation of pure-phased nanoporous perovskite oxides, which not only homogeneously dispersed metal ions but also provided large amounts of heat during the calcination process for the formation of pure-phased perovskite oxides at relatively low temperatures.

This review highlights that nanoporous perovskite oxides usually exhibit better performance than their bulk counterparts in various applications (*e.g.*, gas combustion, soot oxidation, cathodes for SOFCs and metal–air batteries, photocatalytic degradation), which can be ascribed to higher surface areas, more active sites, unique surface properties as well as the rapid diffusion of reactant molecules in the nanopores. For photocatalytic water splitting and CO_2_ oxidation, the crystallinity of catalysts is also very important in relation to their performance. Thus nanoporous perovskite oxide photocatalysts with high crystallinity are still needed for development. In addition, the modification with other metal oxide or metal nanoparticles, doping with other elements, also need to be considered together with the nanoporous structure in design of novel perovskite oxides with excellent performance.

Recently, there is intense interest in the synthesis and applications of *in situ* exsolution of highly active and regenerable nanoparticles on micrometer-sized perovskite oxides, which are usually prepared by calcination and *in situ* reduction at high temperature.^151^–^154^ The combination of nanoporous perovskite oxides as well as *in situ* exsolution of highly active metal nanoparticles on the surface may have interesting catalytic, electronic, and electrochemical properties in a single material. However, the greatest challenge to prepare nanoporous perovskite oxides to support *in situ* exsolution metal nanoparticles is the stability of nanoporous structure at such high calcination temperatures. Even though there are some reports about the *in situ* exsolution of metal nanoparticles on perovskite oxides with several hundreds of nanometers, the nanoporous structures are still not defined enough.^155^,^156^ Growing metal nanoparticles on nanoporous perovskite oxides through electrochemical operation may provide a potential solution for this issue.^157^

## Conflicts of interest

There are no conflicts to declare.
